# Hot isopropanol quenching procedure for automated microtiter plate scale ^13^C-labeling experiments

**DOI:** 10.1186/s12934-022-01806-4

**Published:** 2022-05-09

**Authors:** Jochen Nießer, Moritz Fabian Müller, Jannick Kappelmann, Wolfgang Wiechert, Stephan Noack

**Affiliations:** 1 Institute of Bio- and Geosciences, IBG-1: Biotechnology, Forschungszentrum Jülich GmbH, 52425 Jülich, Germany; 2 Currenta, GmbH & Co. OHG, 51368 Leverkusen, Germany; 3 Computational Systems Biotechnology (AVT.CSB), RWTH Aachen University, 52074 Aachen, Germany

**Keywords:** Lab automation, Isotopic labeling, ^13^C-labeling, Metabolic quenching, Boiling solvent quenching, Isotopically transient experiment, Microbioreactor cultivation, *Corynebacterium glutamicum*

## Abstract

**Background:**

Currently, the generation of genetic diversity for microbial cell factories outpaces the screening of strain variants with omics-based phenotyping methods. Especially isotopic labeling experiments, which constitute techniques aimed at elucidating cellular phenotypes and supporting rational strain design by growing microorganisms on substrates enriched with heavy isotopes, suffer from comparably low throughput and the high cost of labeled substrates.

**Results:**

We present a miniaturized, parallelized, and automated approach to ^13^C-isotopic labeling experiments by establishing and validating a hot isopropanol quenching method on a robotic platform coupled with a microbioreactor cultivation system. This allows for the first time to conduct automated labeling experiments at a microtiter plate scale in up to 48 parallel batches. A further innovation enabled by the automated quenching method is the analysis of free amino acids instead of proteinogenic ones on said microliter scale. Capitalizing on the latter point and as a proof of concept, we present an isotopically instationary labeling experiment in *Corynebacterium glutamicum* ATCC 13032, generating dynamic labeling data of free amino acids in the process.

**Conclusions:**

Our results show that a robotic liquid handler is sufficiently fast to generate informative isotopically transient labeling data. Furthermore, the amount of biomass obtained from a sub-milliliter cultivation in a microbioreactor is adequate for the detection of labeling patterns of free amino acids. Combining the innovations presented in this study, isotopically stationary and instationary automated labeling experiments can be conducted, thus fulfilling the prerequisites for ^13^C-metabolic flux analyses in high-throughput.

**Supplementary Information:**

The online version contains supplementary material available at 10.1186/s12934-022-01806-4.

## Background

Since their inception, isotopic labeling experiments (ILEs) have been applied for various purposes, such as pathway elucidation, model validation, and determination of intracellular flux rates in a host of organisms from bacteria [1] to eukaryotes [2, 3], plants [4–6] and mammalian cell lines [7, 8]. Thus, they have contributed significantly to strain characterization, screening and rational strain development. Over time, they have profited from advancements in analytics [9, 10], modeling [11–13], software development [14–19], and recently automation [20]. Likewise, the experimental techniques became more sophisticated. Replacing its purely stoichiometric predecessor, ^13^C-metabolic flux analysis (MFA) was ab initio a complex method estimating intracellular flux rates by combining extracellular rate measurements, ^13^C-labeling data, a metabolic network model and several computational steps [21]. Still, it was further improved and complicated by moving from steady-state investigations to isotopically dynamic states [22, 23]. Despite considerable progress up to this date, the original cultivation procedure in a bioreactor is commonly still perceived as the gold standard for ILEs. Yet, especially a combination of automation, miniaturization, and parallelization has the potential to alleviate some of the remaining wet laboratory drawbacks [24], i.e., the large amount of expensive labeled substrate required, inflating the cost per experiment and per replicate, the lack of standardization among practitioners, and the comparatively low experimental throughput.

In previous studies, the scale had been decreased to the level of shake flasks [25, 26], mini bioreactors [20] or even to deep well plates [27, 28]. However, when volumes were reduced to the level of mini bioreactors or below, proteinogenic amino acids were analyzed omitting prior quenching of the cellular metabolism. The study by Heux et al. alluded to a quenching method enabling the investigation of free metabolites but did not describe or use said method in the publication. Also, while Heux’s study included automation, their system operated in a volume range of 8–15 mL, whereas the protocols by Ebert et al. and Klinger et al. described manually performed methods within the milliliter range [27, 28].

Furthering this development, in the present study we attempt to address the aforementioned issues by establishing a hot isopropanol quenching method on a robotic cultivation platform to conduct microtiter plate scale, automated isotopic labeling experiments. Capitalizing on the automated quenching procedure, we focused on the analysis of free amino acids, thereby avoiding the requirement of a cultivation time in the order of cellular generation times to reach the stationary labeling state [29, 30] and enabling a response time of minutes rather than hours in ILEs [31]. As a consequence of the miniaturization, the maximum throughput was increased to 48 parallel batches per ILE while the duration and the cost per replicate as well as per experiment were reduced significantly.

## Results and discussion

### Establishing hot solvent quenching on an automated cultivation platform

Historically, many different quenching and extraction [32–36] methods have been developed but the most commonly utilized one today is cold methanol quenching [37, 38]. Even this single term encompasses a range of protocols with varying temperatures, incubation times and methanol solutions. Provided an ultra low temperature device is available, the transfer of such a method to a robotic system is theoretically feasible, albeit with some drawbacks. It would involve impractical pipetting steps with a solvent, inherit the general problem of leakage due to membrane damage during quenching [37, 39], and depend on the toxic solvent methanol.

By instead adapting hot ethanol quenching [40, 41] for an automated platform, the issue of solvent toxicity is sidestepped and additional pipetting actions prone to spilling are avoided since the method utilizes a so-called whole broth sampling approach. Here, the quenching and extraction of metabolites occurs in one step, thus prohibiting the differentiation between endo- and exo-metabolome [39] which on a positive note also renders the issue of metabolite leakage irrelevant. Besides this disadvantage, the method is quite convenient for our robotic setup as the integrated BioShake can heat up to a temperature of 99 °C, exceeding the boiling point of such alcohols as ethanol at 78 °C and isopropanol at 82.5 °C. The limiting factor regarding temperature proved to rather be the low heat transfer coefficient of the commonly used plate material polystyrene. In a pre-experiment, in which a microtiter plate with an isopropanol solution was heated on a BioShake, the liquid’s temperature plateaued at roughly 65 °C.

**Fig. 1:**
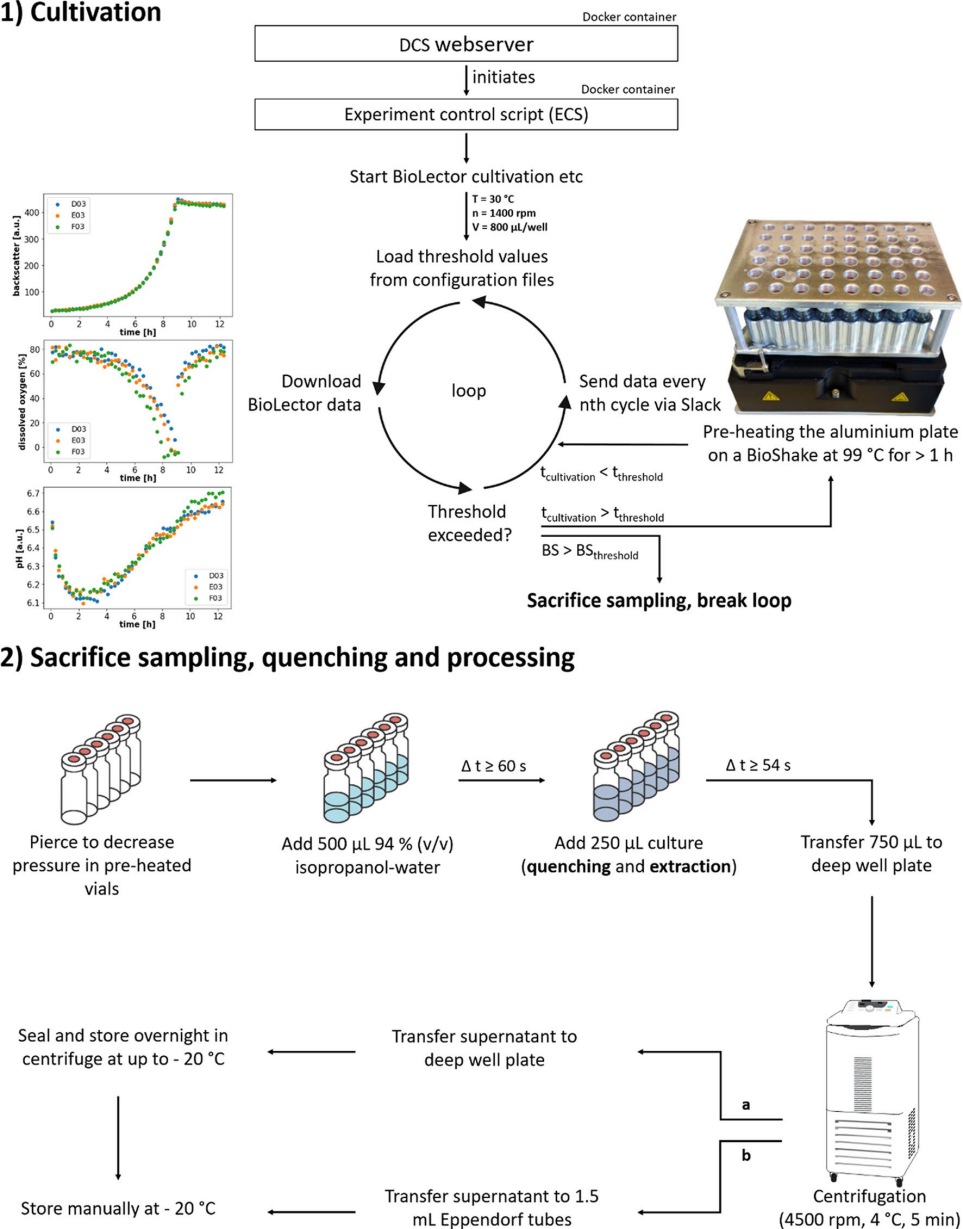
Complete experimental workflow of the automated cultivation, sacrifice sampling, quenching, and sample processing. **1)** A pre-heating step for the hot isopropanol quenching is initiated upon exceeding the cultivation time threshold (t_threshold_), whereas the start of the sampling procedure is dependent on crossing a backscatter threshold (BS_threshold_). **2**) The time for heating the isopropanol solution and the residence time of cellular material inside the heated vials depend on the pipetting scheme of the particular variant of the workflow so the given values are to be understood as lower practical limits. Depending on the subsequent analyses, the extracts may be stored in a sealed deep well plate (**a**) or Eppendorf tubes (**b**).

Due to the increased heat transfer of metals, an aluminum vial holder (Fig. 1) was designed in a 6 × 8 format to be used with 2 mL vitreous vials. Utilizing this plate and the BioShake, the temperature of isopropanol inside vitreous vials could be increased beyond 83 °C (Additional file 1: Fig. S1). Further preliminary tests were performed to investigate the effects of the choice of solvent (Additional file 1: Fig. S2) and of a prolonged incubation at high temperature (Additional file 1: Fig. S3) on the amount of extracted amino acids. In particular, ethanol and isopropanol were considered as commonly used solvents with a sufficiently low boiling point. Longer incubation periods of 20 min in the hot solvent proved to have a minimal effect on the detected amounts for most amino acids. The selection of ethanol or isopropanol did not seem to be of consequence, either, as the observed molar amounts were not consistently higher for one solvent regarding all or even most amino acids. Unlike isopropanol, however, ethanol may be given consideration as a substrate in ILE [42], in which case the addition of an ethanol solution for quenching would at the very least alter the labeling pattern of the ethanol pool. Thus, the setup with isopropanol was henceforth used as described in more detail in the “Methods” section.

Regarding software-wise process control, beyond the use of the vendor software for the robotic system (Freedom EVOware), every experimental run was governed by the in-house developed DigInBio control system (DCS) [43]. The DCS is connected to the laboratory network and devices, features an extensive logging framework, and executes each run within a fresh Docker container using a Python-based experiment control script (ECS). Variables such as the backscatter threshold for triggering sacrifice sampling events (Fig. 1) are defined in separate configuration files and can be adjusted even during the cultivation. In the ECS, the bletl Python package [44] is employed to parse current BioLector data for a given measurement cycle which may be plotted and sent to the practitioner via Slack, e.g., in the format of a heat map portraying the backscatter values of all wells or as line diagrams focusing specifically on selected wells and parameters.

### Validating the quenching method via a spiking experiment

**Fig. 2:**
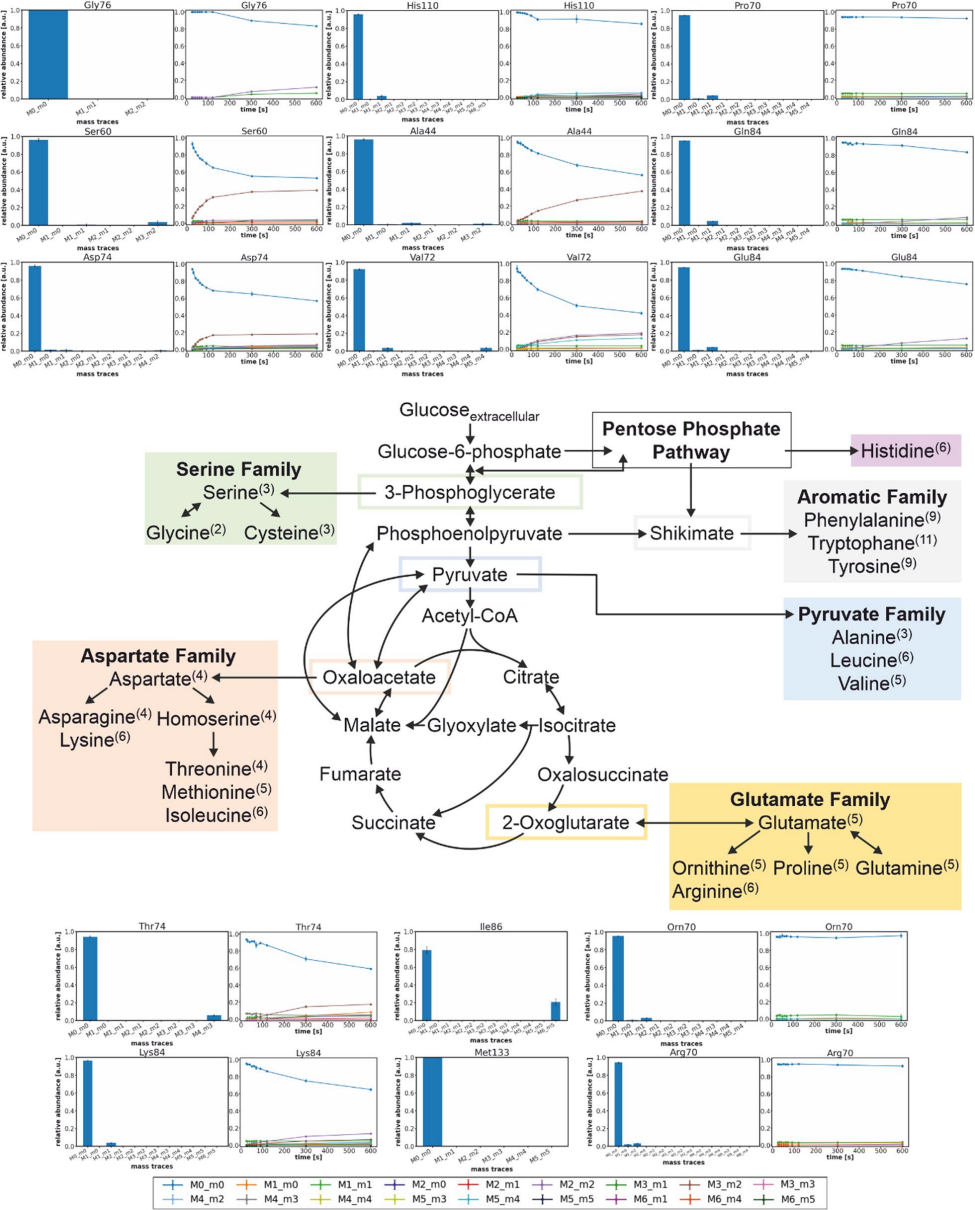
Combined results of the spiking experiment for validation (bar diagrams) and the isotopically instationary ILE as a proof of concept (line diagrams) accompanied by a simplified portrayal of the metabolic network of *C.* *glutamicum* ATCC 13032. The numbers in brackets pertain to each amino acid’s number of carbon atoms. In both experiments, the cultivation began on unlabeled d-glucose but in the former case, U^13^C d-glucose was added to the quenching reagent, and in the latter case, U^13^C d-glucose was pulsed before the transient sampling

Performing a regular cultivation on unlabeled substrate and spiking the quenching reagent with uniformly labeled d-glucose is a reliable method to detect any residual enzyme activity during quenching and has been published previously, albeit in reverse—with fully labeled cells and unlabeled substrate [45, 46]. In the present case (Fig. 2 and Additional file 1: Fig. S4–S8), the amino acids in closest proximity to d-glucose’s entry into the metabolic network of *C. glutamicum*—l-alanine (Ala) and l-serine (Ser)—showed a low single-digit fraction of their uniformly labeled molecular species as expressed in the M3_m2 mass traces of 1.4% and 3.5%, respectively. Separated by a few more reaction steps from pyruvate, for l-valine (Val), too, a 3.5% enrichment for its fully labeled mass trace was observed. This was expected insofar as these amino acids were the most likely to incorporate any labeled material in case of a delay in complete quenching. However, no label was detected for l-glycine (Gly) which is only one reaction removed from Ser. This particular reaction, catalyzed by the serine hydroxymethyltransferase (SHMT), is coupled in an equimolar manner to one carbon metabolism through the co-factor tetrahydrofolate (THF) and by extension to the methylene THF (MTHF) dependent l-methionine (Met) synthesis [47]. On the other end of the connection, Met belonged to the group of amino acids, which were detected with low intensity suggesting a relatively small pool size, but its most intense fragment Met133 showed no full ^13^C-label as well.

Generally, there have been issues detecting some amino acids. Especially for l-tyrosine (Tyr), l-tryptophan (Trp), l-phenylalanine (Phe) and Met, detection could not be guaranteed in every sample. Intuitively, one may surmise that the amount of harvested biomass may be an issue at volume of 800 µL per well, but at the time of sampling biomass values ranged from 8.8 to 9.5 g L^−1^.

Another concern was constituted by the deamidation reactions of Gln to Glu and Asn to Asp which have been reported to occur even at physiological conditions [48]. Hence, one may suspect them to be accelerated under prolonged heat treatment. Since these reactions would influence the detected labeling distributions for Glu and Asp, the effect was investigated under experimental conditions but was found to be negligible (Additional file 1: Fig. S9).

Additionally, the HPLC method could not fully separate l-leucine (Leu) and l-isoleucine (Ile) resulting in a double peak at best. As the present results show, these amino acids must be omitted or at least treated with a great measure of uncertainty when further evaluating the data or using it for ^13^C-MFA.

Surprisingly, the more distant amino acid l-threonine (Thr) showed higher relative quantities of labeled carbon atoms than Ala and Ser. Due to the activity of the phosphotransferase system [49], a high flux towards the synthesis of pyruvate was expected and thus, a faster incorporation of label carbon into Ala than Thr. Therefore, it could be speculated that a combination of a comparatively large pyruvate pool acting as a buffer for Ala and higher flux rates of the enzymes towards Thr synthesis were responsible for these observations. Still, the label enrichment of its two measured fragments remained below 7%. Overall, more than half the measured amino acids and 26 out of 41 fragments were completely unlabeled—excluding natural isotopes. The label incorporation of a further five fragments merely amounted to less than 2% enrichment. This indicates a small bias the impact of which on reaction rates of a fluxome depends on the sensitivity of the reaction in question [21]. While this may affect the accuracy of the measurement, the precision at least can be improved by taking multiple fragments per amino acid into account.

Due to the evident remaining enzyme activity at the onset of quenching, it was hypothesized that the boiling of the solvent, which is inhibited inside the glass vials due to mounting pressure, may be critical to the procedure. Therefore, the same experiment was repeated without vitreous vials in the open wells of the aluminum plate. However, the results turned out nearly identical (Additional file 1: Fig. S10–S13) and the evaporation rates were subject to rather irreproducible position effects so the method with vials was continued.

### Automated isotopically instationary ILE

For this experimental setup, the workflow had to be adapted to accommodate multiple pulsing and sampling events. The delay between the pulse and the onset of quenching had to be as short as possible in order to gain meaningful data of the relevant isotopically instationary period. Since the liquid handler preferably performed a washing step after every pipetting action to regenerate its system air gaps, said delay would have amounted to roughly 57 s meaning a loss of valuable data points particularly for amino acids in close proximity to the substrate d-glucose in the metabolic network. So, instead of harvesting a full column of six wells simultaneously, three liquid handler tips were used for the pulse and the neighboring three for an immediate sacrifice sampling event. This mode of operation decreased the minimal delay to circa 24.5 s. For the next measurements, increasingly long pauses were inserted between pulse and sampling to obtain dynamic labeling data. Performed this way, one may sample up to 16 time points per microtiter plate in biological triplicates.

The dynamic data alone is of limited use when discussing the cellular phenotype as it is influenced both by metabolic pool sizes and fluxes. For example, the slower label enrichment of Ala compared to Ser (Fig. 2) may be due to a larger Ala pool size or to a lower flux value or any combination of the two. Similarly, it seemed intuitive to expect a connection between the stoichiometric biomass requirement of an amino acid and the flux values in its synthesis pathway but the direct transfer to the labeling dynamics is not permissible as the present data shows. Instead, the requirement for Ala (*Y*_Ala/CDW_ = 1.15 mmol g^−1^) is almost fivefold higher than Ser’s (*Y*_Ser/CDW_ = 0.24 mmol g^−1^) [50] which is not reflected in their labeling dynamics. Nonetheless, there are still interesting conclusions to be drawn.

Despite observing a relatively fast time course of labeling for Ser, no transfer of labeled carbon atoms to Gly seemed to occur within one minute of the pulse despite both amino acids being separated merely by a single reaction (Fig. 2). This may hint at a comparatively large pool size of Gly or to a regulation via the abovementioned link to one carbon metabolism and by extension to Met. Unfortunately, Met was not detected in this experiment so this remains a matter of conjecture. It is, however, in accordance with the findings in the spiking experiment in which the mass trace pertaining to fully labeled Ser amounted to 3.5% while Gly remained unlabeled. The slower labeling dynamics of Ala relative to Ser, too, concurred with a lower labeled fraction of about 1.4%.

In the case of both Phe120 and Tyr136 (Additional file 1: Fig. S15), an increasing enrichment of numerous mass traces was detected over time hinting at a scramble of carbon atoms in the pentose phosphate pathway [51, 52]. Additionally, both showed a nearly constant signal in their respective fully labeled mass trace. Since such a fast incorporation of labeled material in less than 25 s seems unlikely judging by previous data [29] and their synthesis pathways encompass ten reactions from phosphoenolpyruvate, this might be an interfering signal which would explain the similarly unexpected observations in the spiking experiment. Even the enrichments of the fully labeled mass traces of Phe120 amounted to similar values of 18.3% in the spiking experiment and between 20 and 25% in the present data. However, only in the spectra of fully labeled Phe an unexpected signal with an m/z of 129.1025 was found aside from the expected fully labeled species of fragment Phe120 with an m/z ratio of about 128 [53].

Regarding the reconcilability of labeling dynamics of such amino acids, which are derived from others, the two nodes of l-aspartate (Asp) and l-glutamate (Glu) were of most interest (Fig. 2). For Asp, a relatively fast labeling time course was observed implying a smaller pool size compared to Ala and/or higher flux rates since the Asp synthesis required additional reaction steps. In accordance with expectations, its derived amino acids l-homoserine (Hom), Thr, and l-lysine (Lys) each showed—compliant with expectations—a less inclined variant of Asp’s labeling curve.

It was hypothesized before that “large buffer pools for transamination involving α-keto acids” [29], referring to the Glu pool in first and the l-glutamine (Gln) pool in second instance, were responsible for the delayed labeling of TCA cycle intermediates and the amino acids themselves. In the cited publication, the Glu data was not included due to measurement noise but the present data strongly supports the hypothesis. Judging by the Glu130 curve, a minor incorporation of label into Glu had barely occurred at the onset of measurements after 24.5 s but the decrease of M0_m0 seems more linear and slower in contrast to the initially parabolic one of e.g. Ser, Asp, Ala, and others (Fig. 2 and Additional file 1: Fig. S14–S18). Gln showed a similar trend, merely offset by more than two minutes. Even after five minutes, the enrichment of each Gln fragment’s unlabeled mass trace was only decreased by a few percent. Combined with the observation that the amino acids “downstream” from Glu and Gln, namely l-arginine (Arg), l-proline (Pro), and l-ornithine (Orn), remained unlabeled during the sampled time frame of 10 min, it stands to reason that the large Glu pool indeed acted as a buffer for several minutes and the Gln pool as a secondary one delaying the spread of ^13^C-isotopes.

In summary, the established automated workflow’s capacity to conduct ILEs has been demonstrated successfully for most observed free amino acids. Especially the aim of increasing the throughput while decreasing time and monetary investments was achieved. The automated quenching method was designed for the analysis of amino acids, but may be expanded to other metabolites such as sugar phosphates. In that case it has to be investigated whether the inactivation of enzymes occurs sufficiently fast for the expectable higher turnover rates and a faster onset of label incorporation in these metabolites [38] and whether the prolonged heat treatment decreases their concentration below the limit of detection.

Regarding the applicability of this method to ^13^C-metabolic flux analysis in particular, the brief window of residual activity observed during quenching may slightly alter the labeling pattern of some free amino acids, thereby possibly influencing the fluxome. In addition to labeling data, the determination of extracellular flux rates constitutes a prerequisite for MFA. Since backscatter and dissolved oxygen are measured online in the BioLector and at-line assays are well established for further analysis of transient samples [54], the specific growth rate as well as substrate and (by)-product formation rates are readily accessible via bioprocess modeling [55]. Due to the lack of an off-gas analysis in this setup, the carbon dioxide evolution rate (CER) is not known the impact of which on the sensitivity of a MFA and statistical identifiability of the network needs to be investigated. Depending on the cultivation conditions, though, even in a bioreactor the correlation between off-gas CO_2_ content and CER can be uncertain or misleading due to the pH-dependent equilibrium of bicarbonate and CO_2_ in the medium [56, 57].

## Conclusions

An automated hot isopropanol quenching method has been established and experimentally validated with the Gram-positive wild-type organism *C. glutamicum* ATCC 13032 for the qualitative analysis of free amino acids. Using this method within the framework of a robotic platform and a microbioreactor cultivation system, an automated ILE was successfully conducted at a microtiter plate-scale and transient labeling data was generated. Implicitly, it was shown that the biomass in said small-scale cultivation with a volume of 800 µL per well is sufficient for LC–MS/MS analyses of most free amino acids. Furthermore, some lingering problems of ILEs, namely the high substrate cost and time investment as well as the low experimental throughput, were addressed, enabling ILEs with up to 48 batches in a single day. Due to the high degree of parallelization and the facility for transient sampling, the performance of parallel labeling experiments with different tracer mixtures sampled at several time points including an isotopically stationary endpoint was enabled. Finally, an automated method may harness the benefits of digitization, allowing the integration of any laboratory device into a laboratory network, providing immediate data evaluation during automated experiments and interfacing with cloud infrastructure.

## Methods

### Strain and cultivation conditions

The Gram-positive biotin-auxotrophic wild-type strain *C. glutamicum* ATCC 13032 was used in all experiments of this study. In every pre- and main culture, CGXII medium [58] with 2% (w v^−1^) d-glucose was employed. Pre-cultures were performed over-night at 300 rpm and 30 °C in 500 mL 4-baffled shaking flasks with 10% filling volume. Main cultures were cultivated in 6 × 8 FlowerPlates in a BioLector Pro system (Beckman Coulter GmbH, Baesweiler, Germany) with filling volumes of 800 µL per well at 1400 rpm and 30 °C. Biomass was continuously monitored in 5 min cycles via on-line backscatter measurements with a gain of 3. Since FlowerPlates without optodes were utilized, dissolved oxygen and pH were not measured in this case but can be for future applications. A Tecan Freedom EVO robotic system (Tecan Deutschland GmbH, Crailsheim, Germany) with a liquid handling arm with eight steel tips and a robotic manipulator arm as well as access to a Hettich Rotanta 460 Robotic centrifuge (Andreas Hettich GmbH & Co. KG, Tuttlingen, Germany) and a BioShake 3000 elm (QInstruments GmbH, Jena, Germany) was tasked with automated liquid handling and sample processing.

### Automated sampling and hot isopropanol quenching

The automated sacrifice sampling events were triggered by a backscatter threshold selected to be reached during the mid-exponential growth phase of the cultivation. Coupled to a cultivation time threshold, a 6 × 8 aluminum plate equipped with closed 2 mL vitreous vials was pre-heated on a BioShake at least an hour before sampling, using its highest temperature setting of 99 °C. After the backscatter threshold was exceeded in any well, air was extracted from the vials to decrease pressure and 500 µL quenching reagent (94% (v v^−1^) isopropanol-water) were administered to each vial. Subsequently, columns of up to six cultivation wells were harvested simultaneously and 250 µL culture broth were dispensed directly into the hot isopropanol solutions in order to achieve a concomitant quenching of the cellular metabolism and extraction of intra- and extracellular amino acids. The extracts were then transferred to a deep well plate for a 5 min centrifugation step at 4500 rpm and 4 °C. Finally, the supernatants were dispensed in 1.5 mL Eppendorf tubes and stored at − 20 °C. To emphasize, the mere manual steps of the main experiment consisted of media preparation, inoculation of the main culture, supplying the robot deck with the aforementioned plates and storing the samples at the end of the experiment. Depending on the starting OD_600_, the desired growth phase, media composition, and strain, the duration of an experiment may vary but in this study all experiments were conducted in a single work day each.

### LC–MS/MS analysis of amino acid mass traces

Undiluted cell-free extracts were analyzed using an Agilent 1260 Infinity II liquid chromatography system (Agilent Technologies, Waldbronn, Germany) coupled with a Sciex TripleTOF 6600 (AB Sciex Germany GmbH, Darmstadt, Germany) mass spectrometer endowed with a Turbolon spray ion source. All samples were spiked 1:2 with a 1:4000 dilution of a U^13^C-U^15^N-labeled cell free amino acid mixture from Sigma-Aldrich (Sigma-Aldrich Chemie GmbH, D-89555 Steinheim, Germany) as an internal standard. A 150 mm × 2 mm Phenomenex Luna SCX column (Phenomenex Ltd., Aschaffenburg, Germany) with a pore size of 100 Å and a particle size of 5 µm was used to separate the extracted amino acids before MS analysis. Further details on the LC method have been described previously [57]. MS parameters are relayed in the supplement (Additional file 1: Table S1). Feature recognition was performed via the MultiQuant Software by Sciex and the calculation of tandem mass isotopomer distributions (TMIDs) was conducted in Python.

### Validation of the quenching procedure via a spiking experiment

The main cultivation on unlabeled d-glucose and the quenching process were performed as described above in 12 biological replicates. However, 0.2 g L^−1^ U^13^C d-glucose were added to the quenching reagent to monitor residual enzyme activity during the quenching procedure via the enrichment of labeled carbon atoms in the extracted free amino acids.

### Automated isotopically instationary ILE

An ILE was conducted to obtain transient labeling data. Here, the main cultivation was initiated with a decreased starting volume of 750 µL and a 50 µL pulse of 80 g L^−1^ U^13^C d-glucose was administered directly before sampling during the mid-exponential growth phase. For the purpose of this proof of concept, ten time points were selected for sacrifice sampling in biological triplicates with delays between pulse and onset of quenching of 25 s, 30 s, 40 s, 50 s, 60 s, 70 s, 90 s, 120 s, 300 s, and 600 s. Based on a prior publication, the most significant changes in the labeling of amino acids adjacent to glycolysis and pentose phosphate pathway (PPP) were expected to occur within the first minute after the pulse wherefore the sampling time points were concentrated around this time frame [29].

### Nomenclature of amino acid MS/MS fragments

In the present study, l-amino acid fragments analyzed via LC–MS/MS are referred to by the amino acid three letter code followed by the m/z ratio of the observed fragment, e.g., Tyr136 for the product ion of l-tyrosine with the m/z ratio of 136.

## Supplementary Information

**Additional file 1. **Additional methods, Table S1 and Figures S1–S18.

## Data Availability

The datasets used and analyzed during the current study are available from the corresponding authors on reasonable request.

## Abbreviations

CER: Carbon dioxide evolution rate
CTR: Carbon dioxide transfer rate
*C. glutamicum*: *Corynebacterium glutamicum*
DCS: DigInBio control system
ECS: Experimental control script
ILE: Isotopic labeling experiment
MFA: Metabolic flux analysis
(M)THF: (Methylene) tetrahydrofolate
PPP: Pentose phosphate pathway
SHMT: Serine hydroxymethyltransferase
TCA cycle: Tricarboxylic acid cycle
