# Supplementary material for: Hot isopropanol quenching procedure for automated microtiter plate scale 13C-labeling experiments
Source: Microb Cell Fact. 2022 May 9;21:78. doi: 10.1186/s12934-022-01806-4 (PMC9082905; doi:10.1186/s12934-022-01806-4)
Supplement: Supplementary file 1 — Additional file 1. Additional methods, Table S1 and Figures S1–S18. [file 12934_2022_1806_MOESM1_ESM.docx]

**Additional file 1**

**Hot isopropanol quenching procedure for automated microtiter plate scale ^13^C-labeling experiments**

Jochen Nießer^1^, Moritz Fabian Müller^1^, Jannick Kappelmann^1,2^, Wolfgang Wiechert^1,3^, Stephan Noack^1#^

# **Additional methods**

**MS parameters**

**Table S1.** MS parameters used for the LC‑MS/MS analysis of amino acid extracts generated with hot isopropanol quenching.

| **MS parameter** | **value** |
| --- | --- |
| Collisionally activated dissociation (CAD) gas [psi] | 5 |
| Curtain gas flow [psi] | 25 |
| Ion source gas 1 [psi] | 45 |
| Ion source gas 2 [psi] | 65 |
| IonSpray voltage [V] | 5500 |
| Ion source temperature [°C] | 650 |


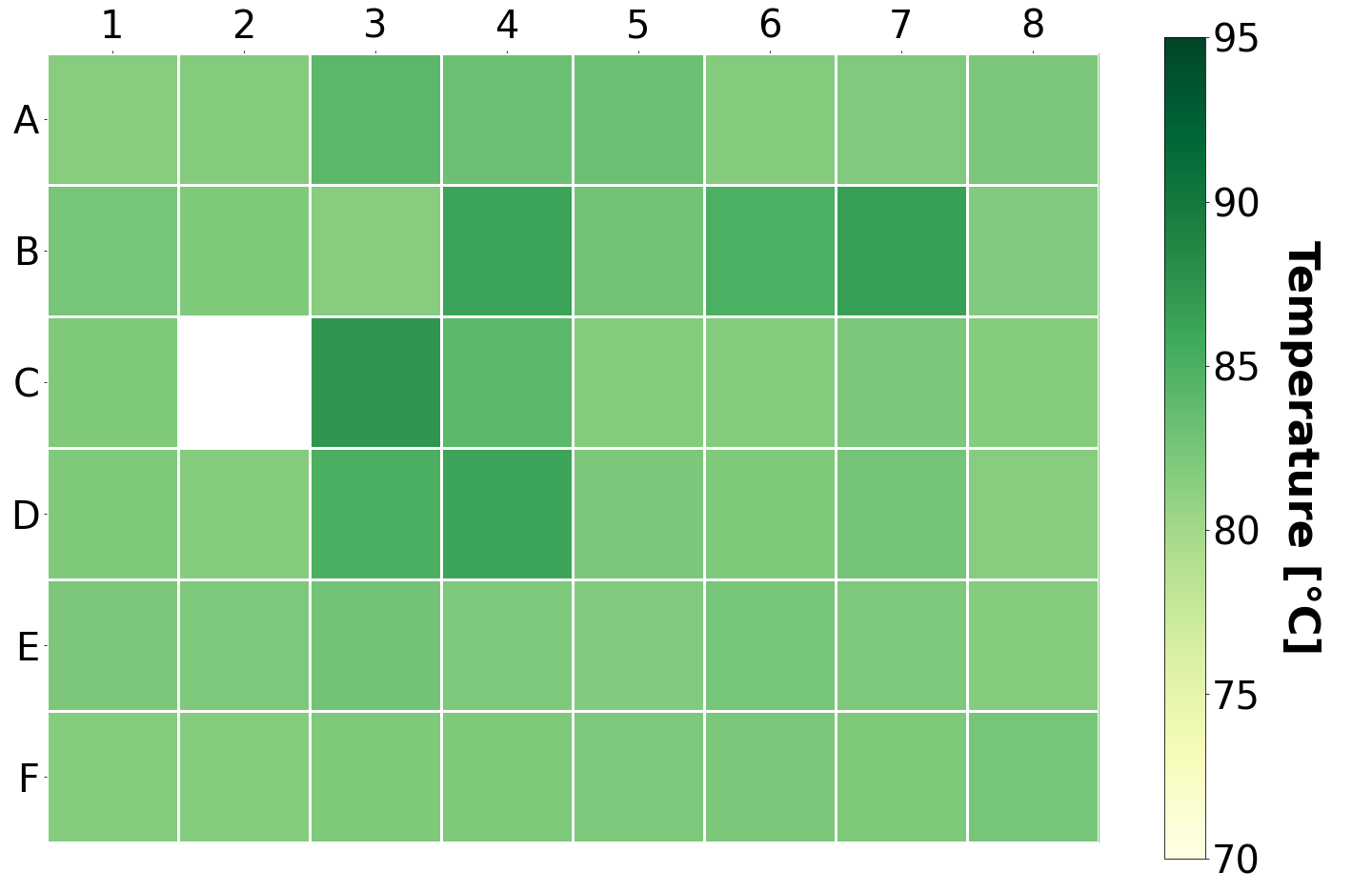
**Heat distribution along the aluminum plate**

**Figure S1.** 500 µL of a 94 % (v/v) isopropanol‑water solution were transferred into pre‑heated vials which were placed inside the aluminum plate on a BioShake set to 99 °C. The temperature of the liquid phase was measured with a digital thermometer after 60 s incubation time.

**Choice of solvent for quenching**

In a pre‑experiment, 250 µL of an overnight culture of *C. glutamicum* ATCC 13032 grown on CGXII medium with 4 % d‑glucose were injected into 500 µL of either hot ethanol or hot isopropanol in closed vitreous vials inside the 6x8 aluminum plate (Fig. 1). For the treatment with ethanol, six biological replicates were cultivated, and a further three for the treatment with isopropanol. After 20 min incubation at a BioShake temperature setting of 99 °C, the extracts were centrifuged at 13000 rpm for 5 min and the supernatants analyzed via LC‑MS/MS.

As can be seen in Figure S2, the differences between the treatment with ethanol and isopropanol appear to be dependent on the amino acid but a structural disparity suggesting a more effective permeabilization of the cell membrane by either one solvent was not observed. A paired two‑sided Student’s t‑test was performed with a significance level of 5 % to compare the amino acid amounts yielded from whole‑broth sampling with ethanol and isopropanol but significant differences were only observed for seven amino acids. Among these, higher amounts for Asn, Trp, Tyr, Arg, and His were observed in the isopropanol group and for Thr and Ala in the ethanol group.


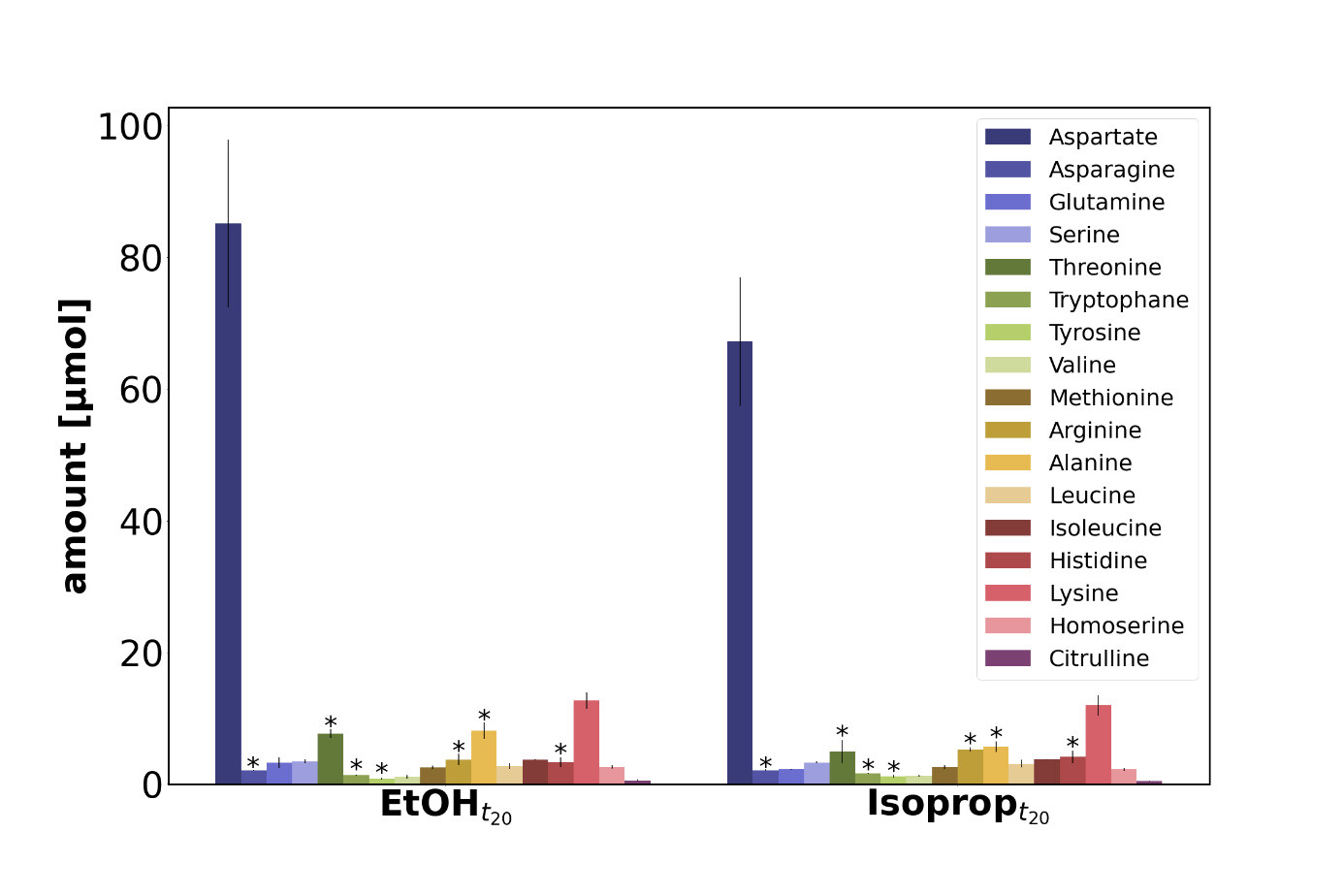


**Figure S2.** Comparison of amino acid amounts obtained by quenching and extraction with either hot ethanol or isopropanol solutions. The extracts were incubated on a heated BioShake set to 99 °C for 20 min. Significant differences between the ethanol and isopropanol group calculated via a paired two‑sided Student’s t‑test with a significance level of 5 % were indicated with (*) over the corresponding bar.

Figure S3 portrays the difference in amounts between a sample taken shortly after quenching with either ethanol or isopropanol and those taken after an incubation time of 20 min (Fig. S2) in order to investigate the influence of prolonged heat on the amino acid yield. Since the method is applied in a strictly qualitative manner to detect labeling distributions, a reduction would only be problematic if the amount of a given amino acids is reduced below the limit of detection of the LC‑MS/MS.


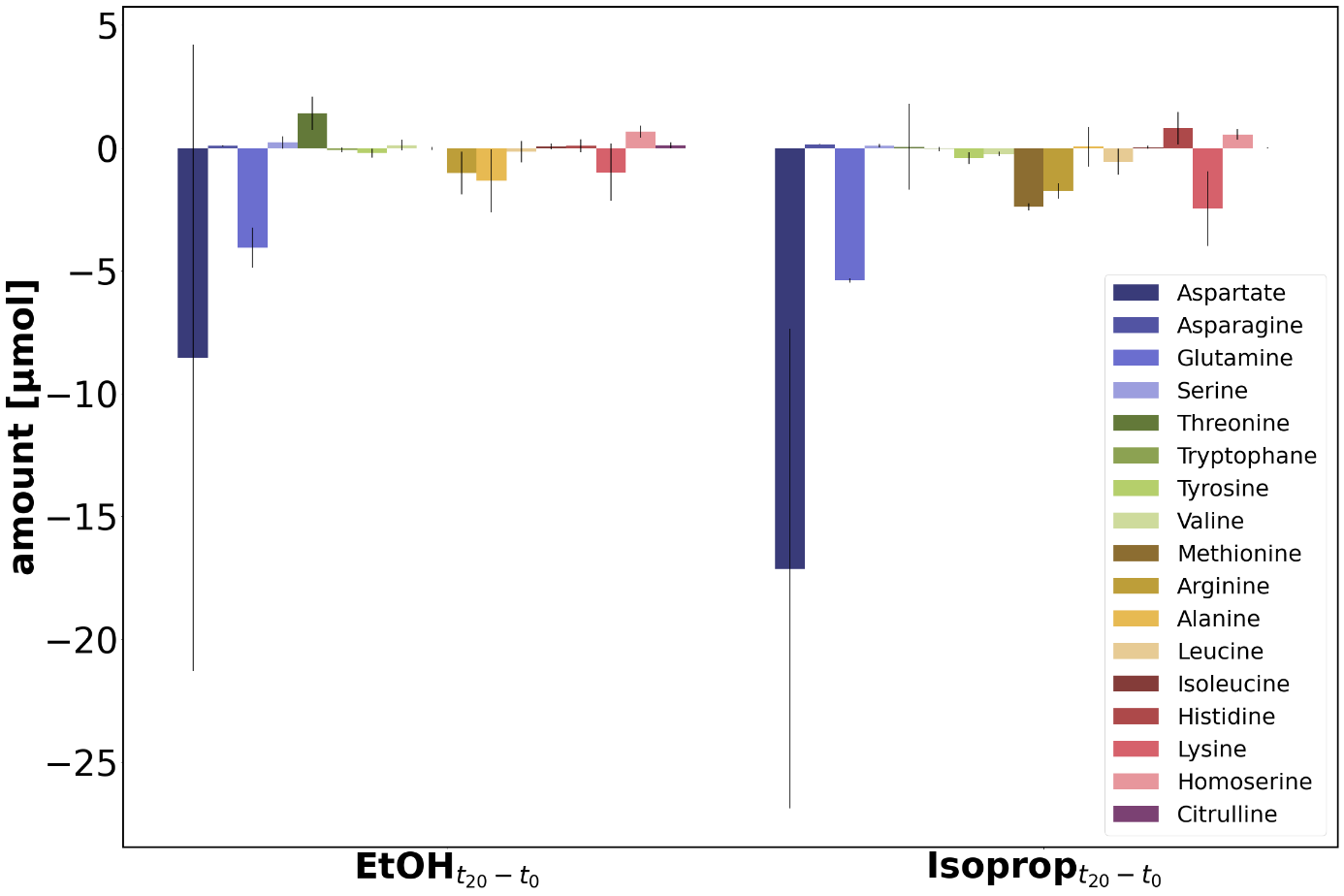


**Figure S3.** Differences of amino acid amounts obtained by quenching and extraction with either hot ethanol or isopropanol solutions. The extracts were each incubated on a heated BioShake set to 99 °C and sampled directly and after 20 min of incubation to test the influence of prolonged heat on the amino acid yield.

**Validation of the quenching procedure via a spiking experiment**

The following figures show the tandem mass isotopomers distributions of all detected fragments paired with exemplary extracted ion chromatograms.

**
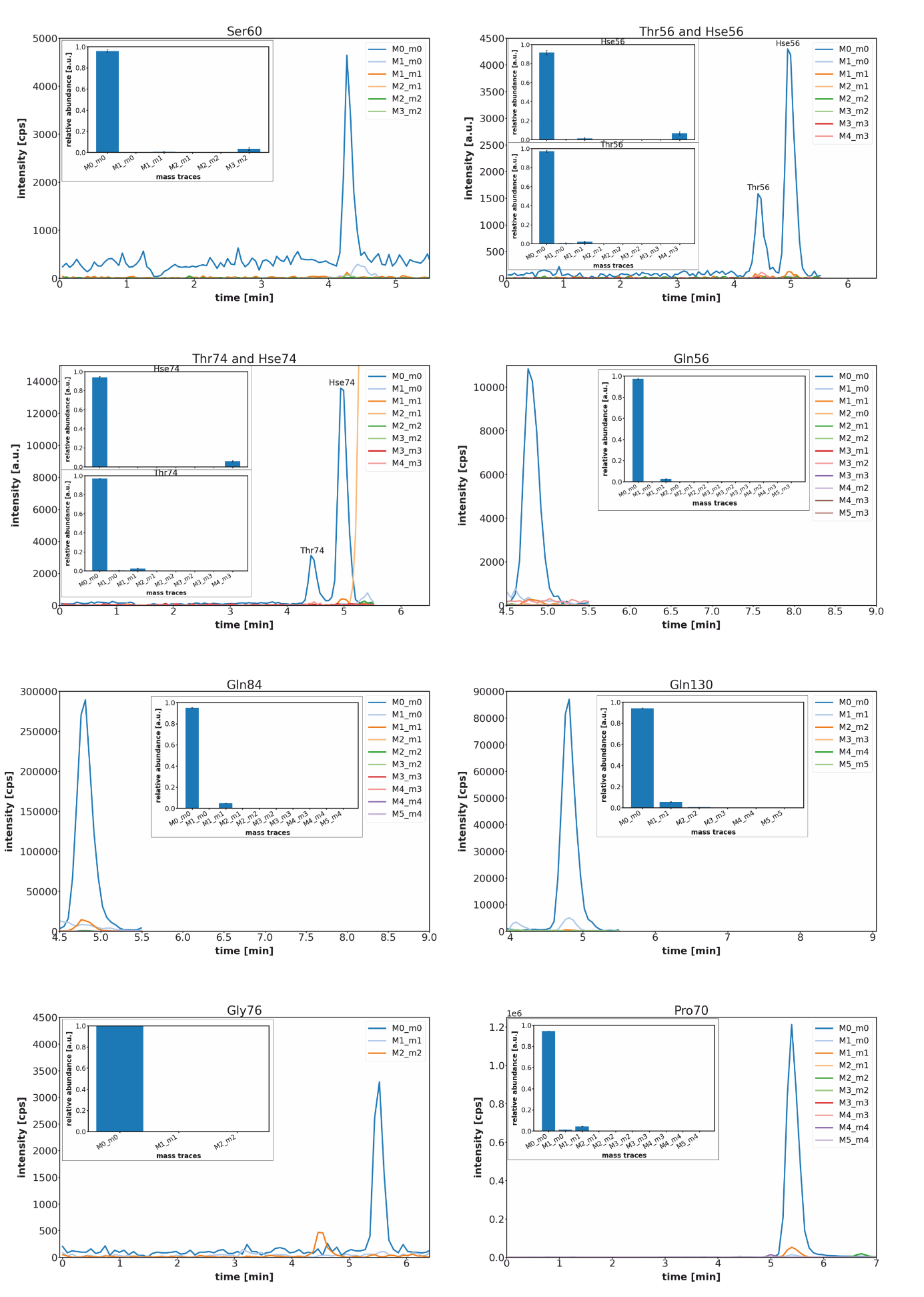
Figure S4.** Average TMIDs across twelve biological replicates and exemplary MS/MS spectra of amino acids with hydrophobic side chains Ser, Hse, Thr, and Gln and the small amino acids Gly and Pro. The data was obtained during the spiking experiment to validate hot isopropanol quenching*.*


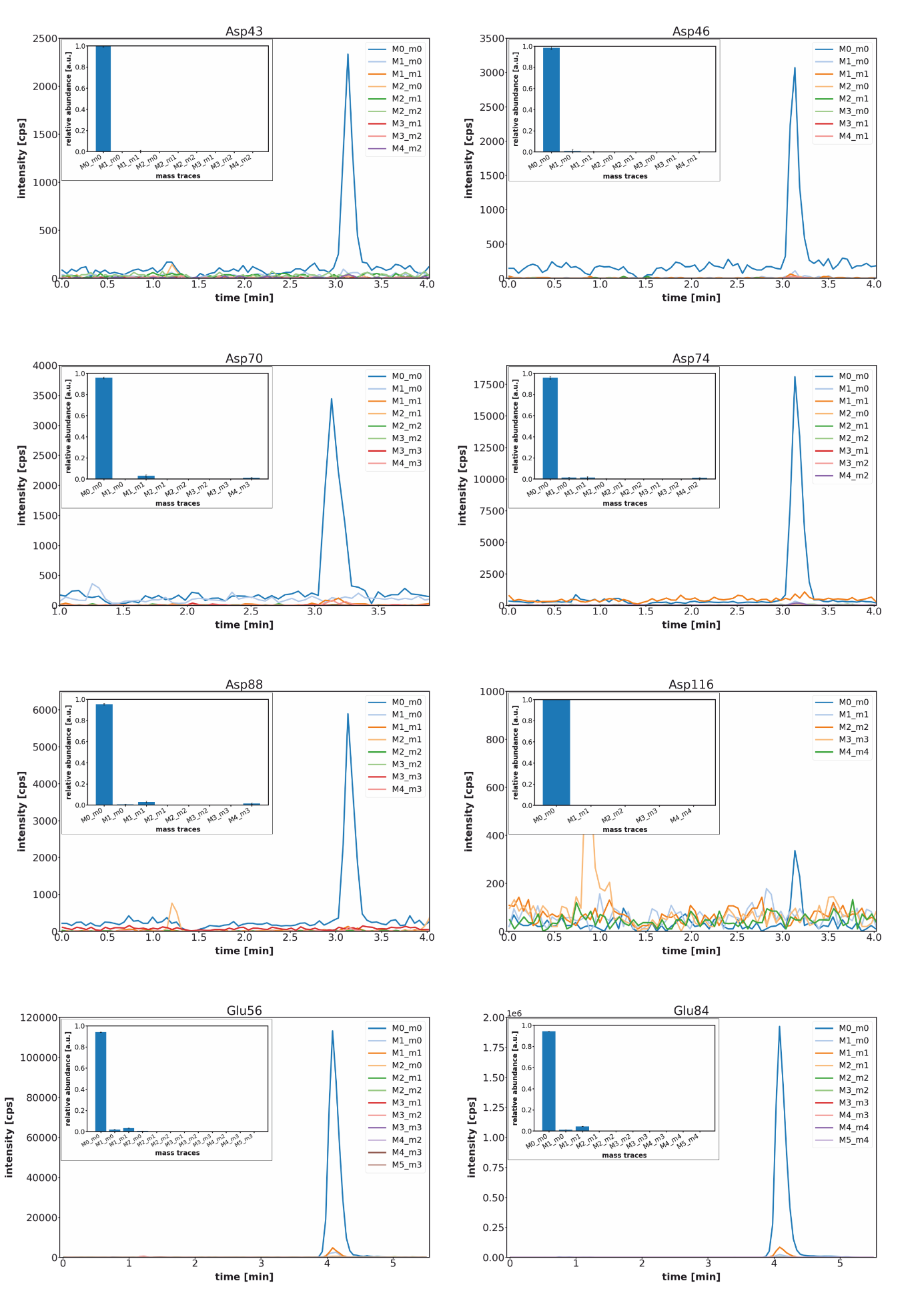
**Figure S5.** Average TMIDs across twelve biological replicates and exemplary MS/MS spectra of amino acids with acidic side chains Asp and Glu. The data was obtained during the spiking experiment to validate hot isopropanol quenching*.*


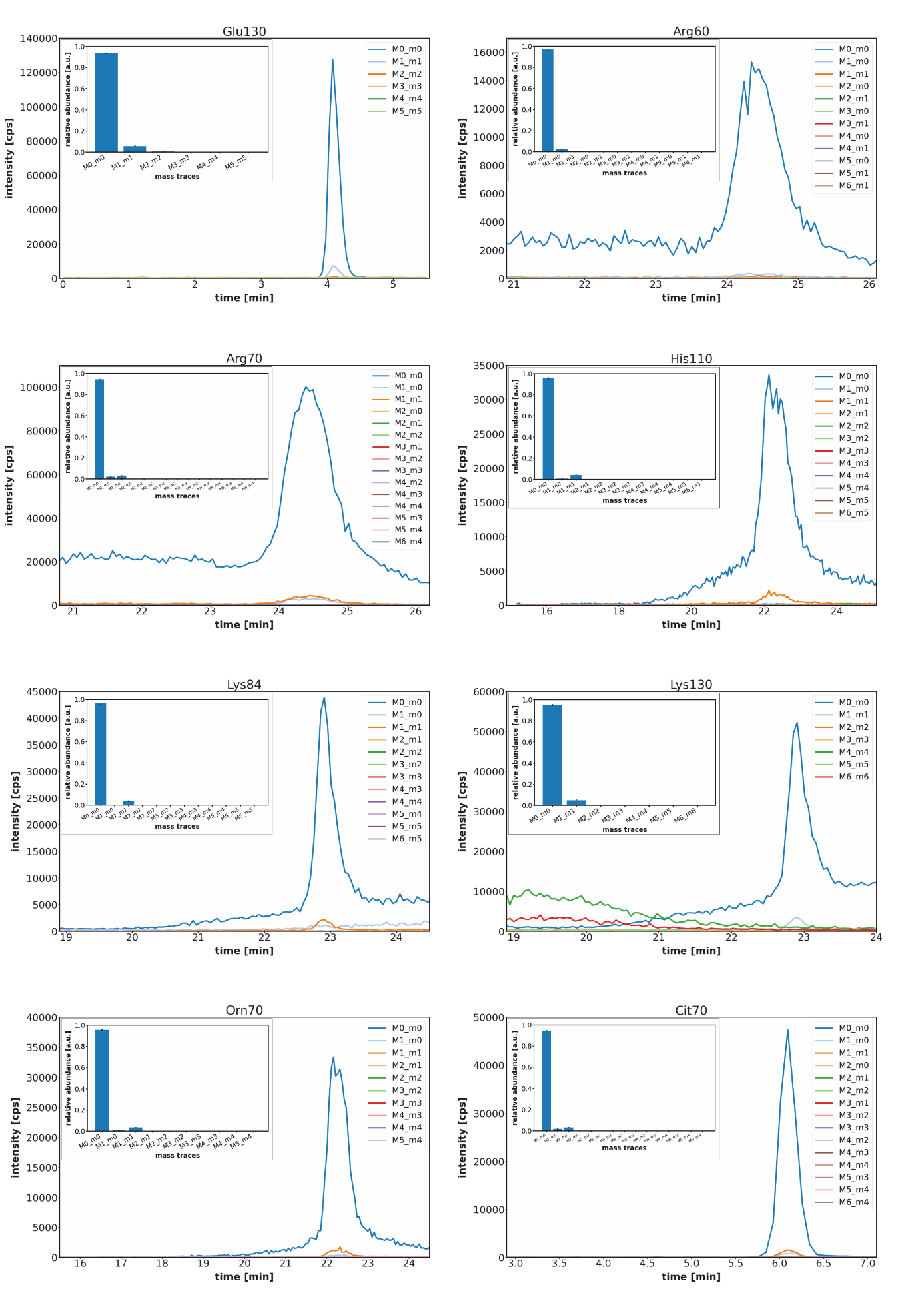
**Figure S6.** Average TMIDs across twelve biological replicates and exemplary MS/MS spectra of Glu as well as the amino acids with basic side chains Arg, His, Lys, Orn, and Cit. The data was obtained during the spiking experiment to validate hot isopropanol quenching.


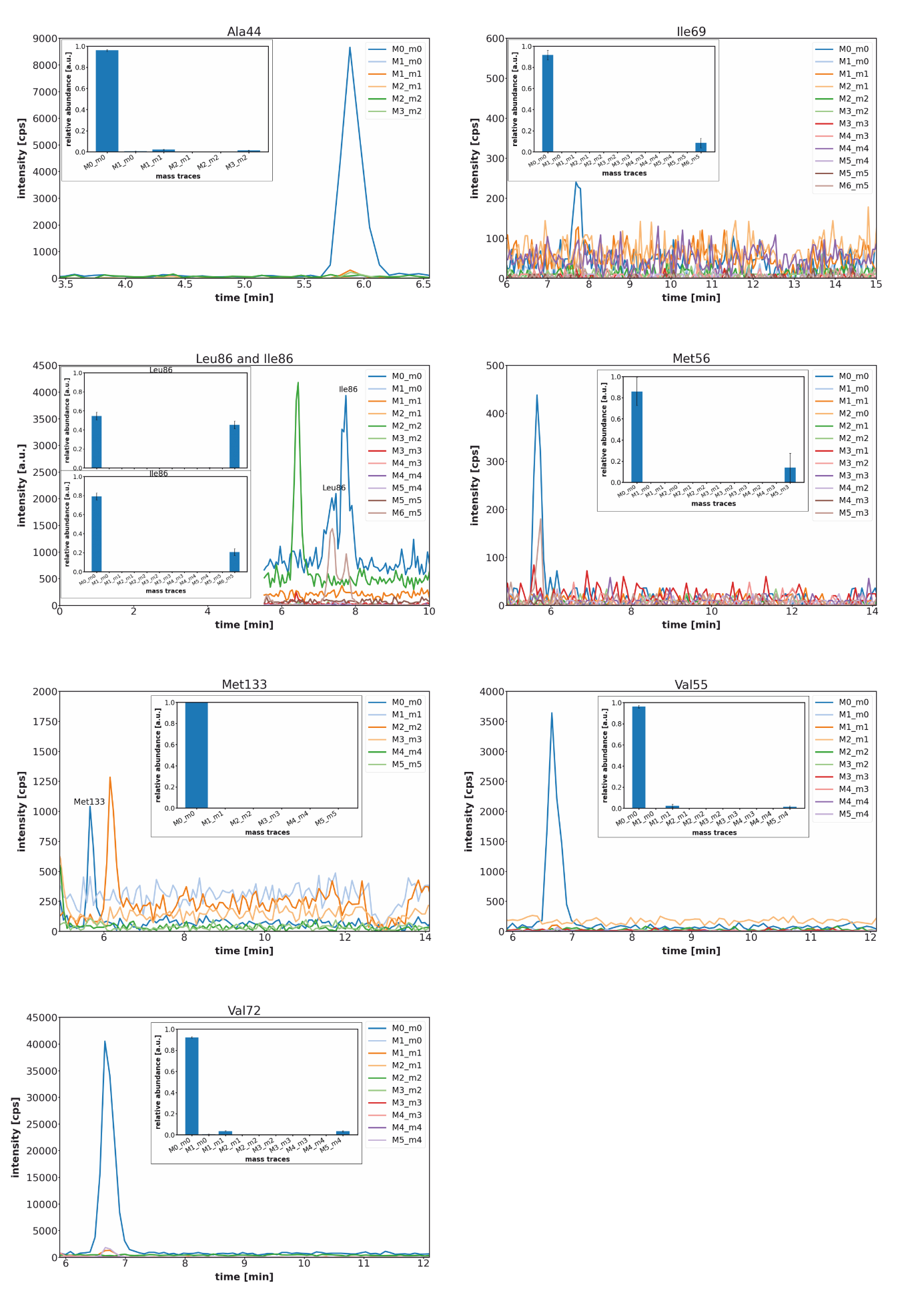
**Figure S7.** Average TMIDs across twelve biological replicates and exemplary MS/MS spectra of amino acids with polar neutral side chains Ala, Ile, Leu, Met, and Val. The data was obtained during the spiking experiment to validate hot isopropanol quenching.


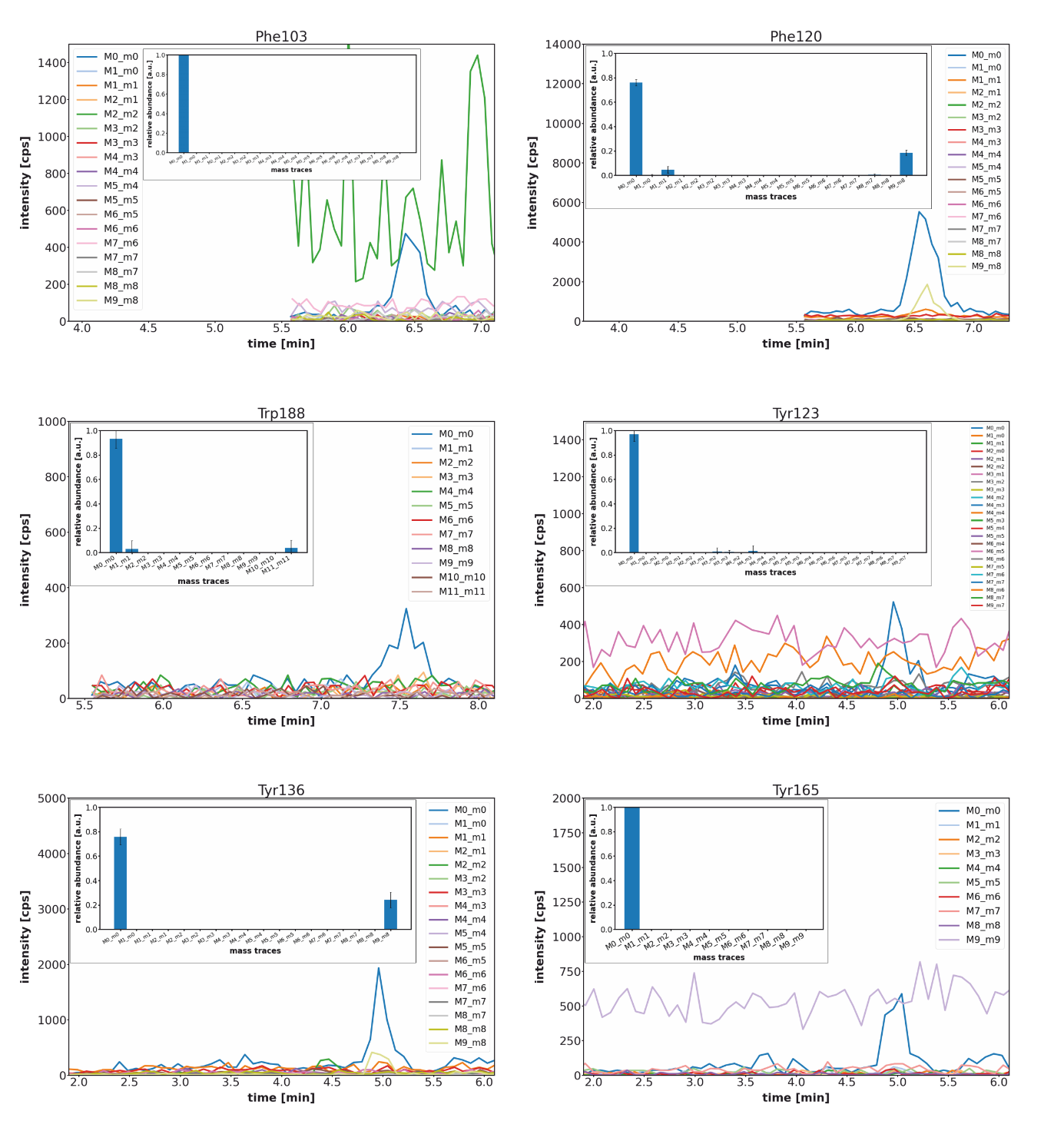
**Figure S8.** Average TMIDs across twelve biological replicates and exemplary MS/MS spectra of Glu as well as the amino acids with aromatic side chains Phe, Trp, and Tyr. The data was obtained during the spiking experiment to validate hot isopropanol quenching.

**Investigating the deamidation reaction of Asn and Gln**


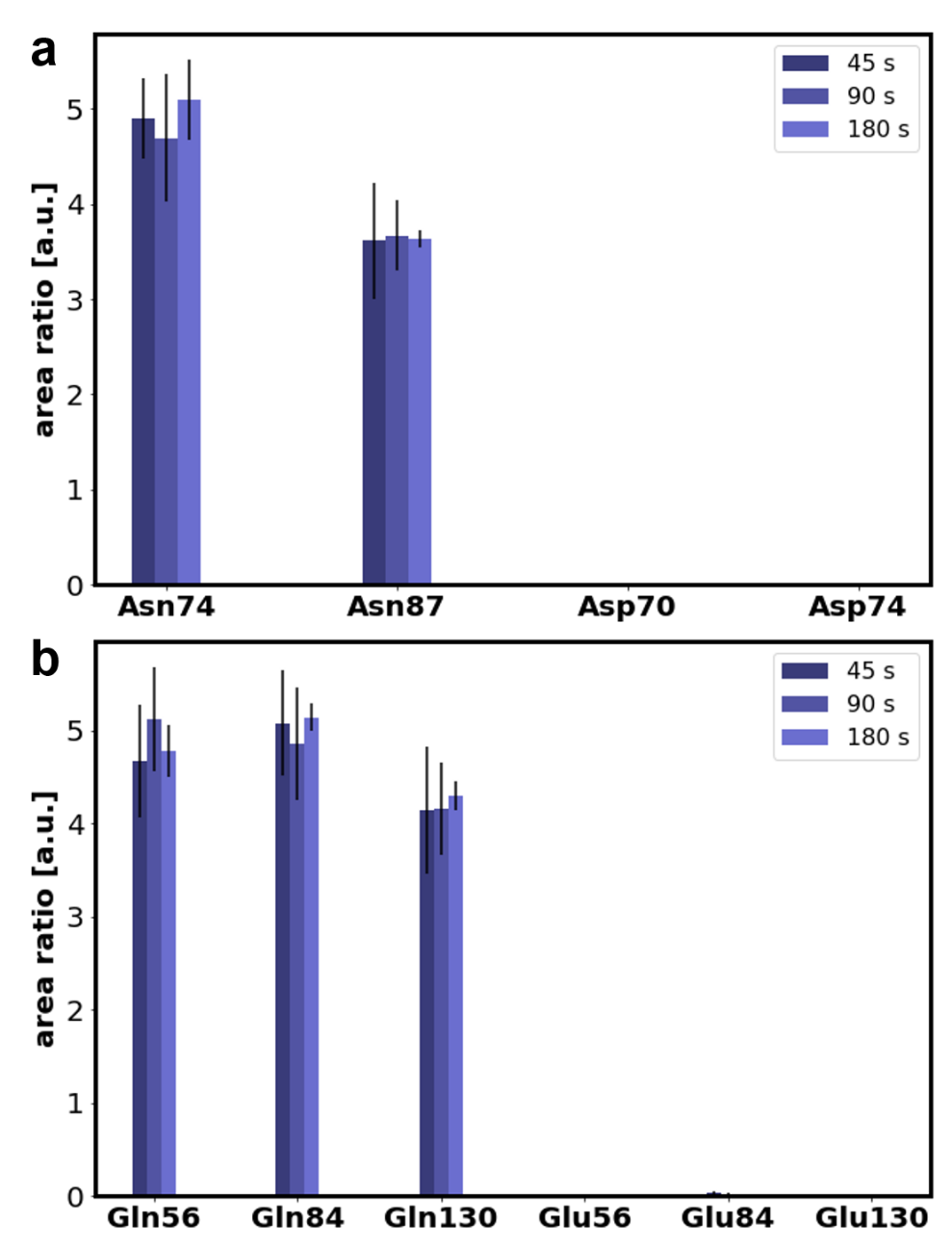
To investigate the extent to which the deamidation reactions of Asn and Gln to Asp and Glu, respectively, occurred during hot isopropanol quenching, the automated workflow as shown in Fig. 1 and described in the methods section was performed with a mixture containing Asn and Gln at a concentration of 1.67 mM, each. Incubation times of 45 s, 90 s, and 180 s inside the heated vials were tested with three replicates each. An internal standard was administered before LC‑MS/MS analysis as stated in the methods section and the final dilution of the samples amounted to 1:120. As portrayed in Figure S9, no Asp was detected and only traces of Glu were observed with intensities which were orders of magnitude below those of Gln’s fragments. The incubation times relevant to the workflow were not found to be of significant influence, either, which was confirmed via a paired two‑sided Student’s t‑test with a significance level of 5 %.

**Figure S9.** Area ratio of fragments of (a) Asn and Asp and (b) Gln and Glu after conducting the automated quenching workflow with incubation times inside the heated vitreous vials of 45 s, 90 s, and 180 s.

**Spiking experiment in open wells**

As referred to in the main text, the spiking experiment, where cells were grown on unlabeled d‑glucose and U^13^C d‑glucose was added to the quenching reagent, was repeated using open wells meaning the quenching occured directly inside the wells of the aluminum plate. The results are portrayed in Fig. S10 ‑ S13.


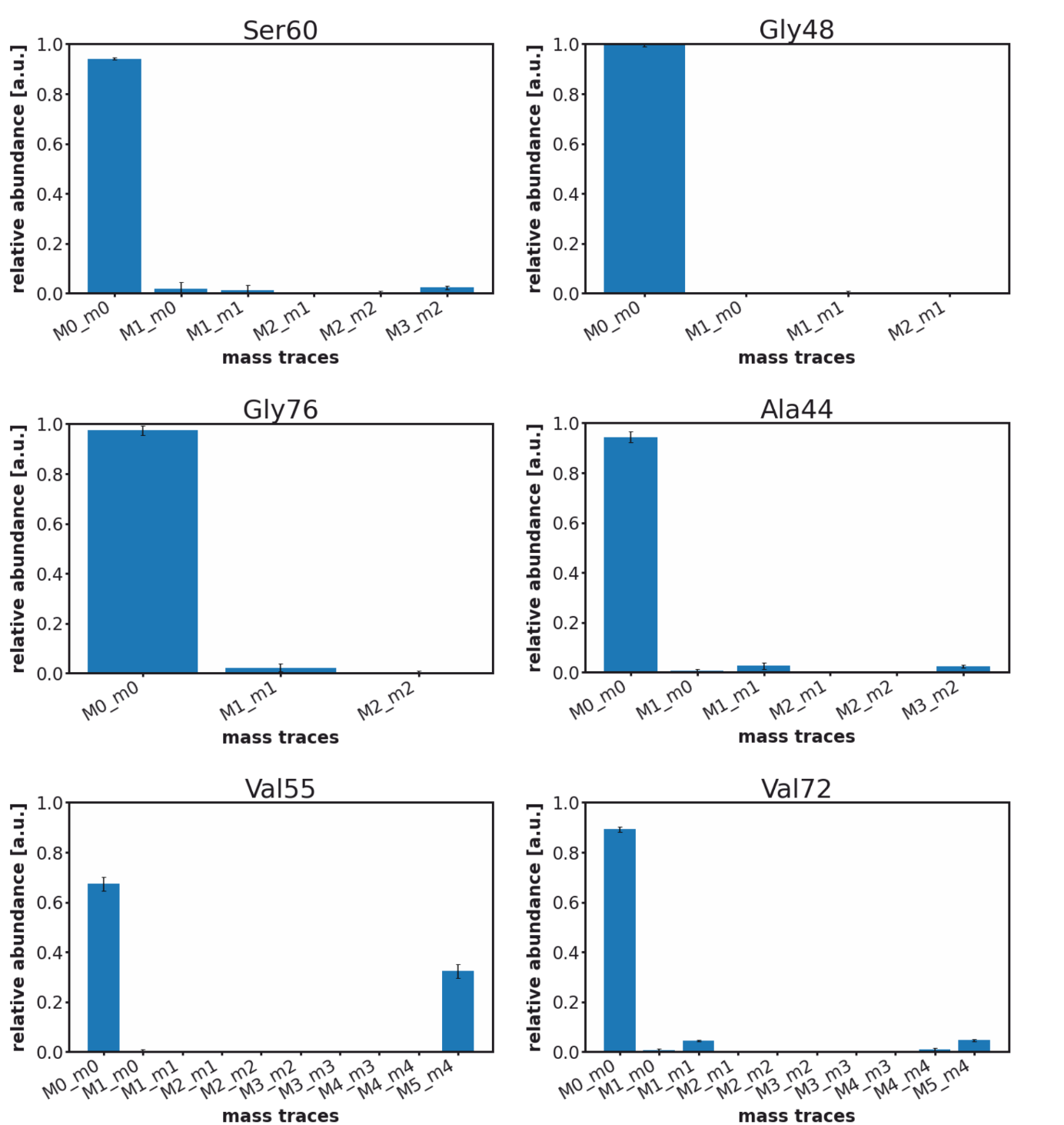


**Figure S10.** Average TMIDs across twelve biological replicates resulting from the spiking experiment in open wells. Here, the measured amino acids from the Ser and pyruvate families are potrayed.


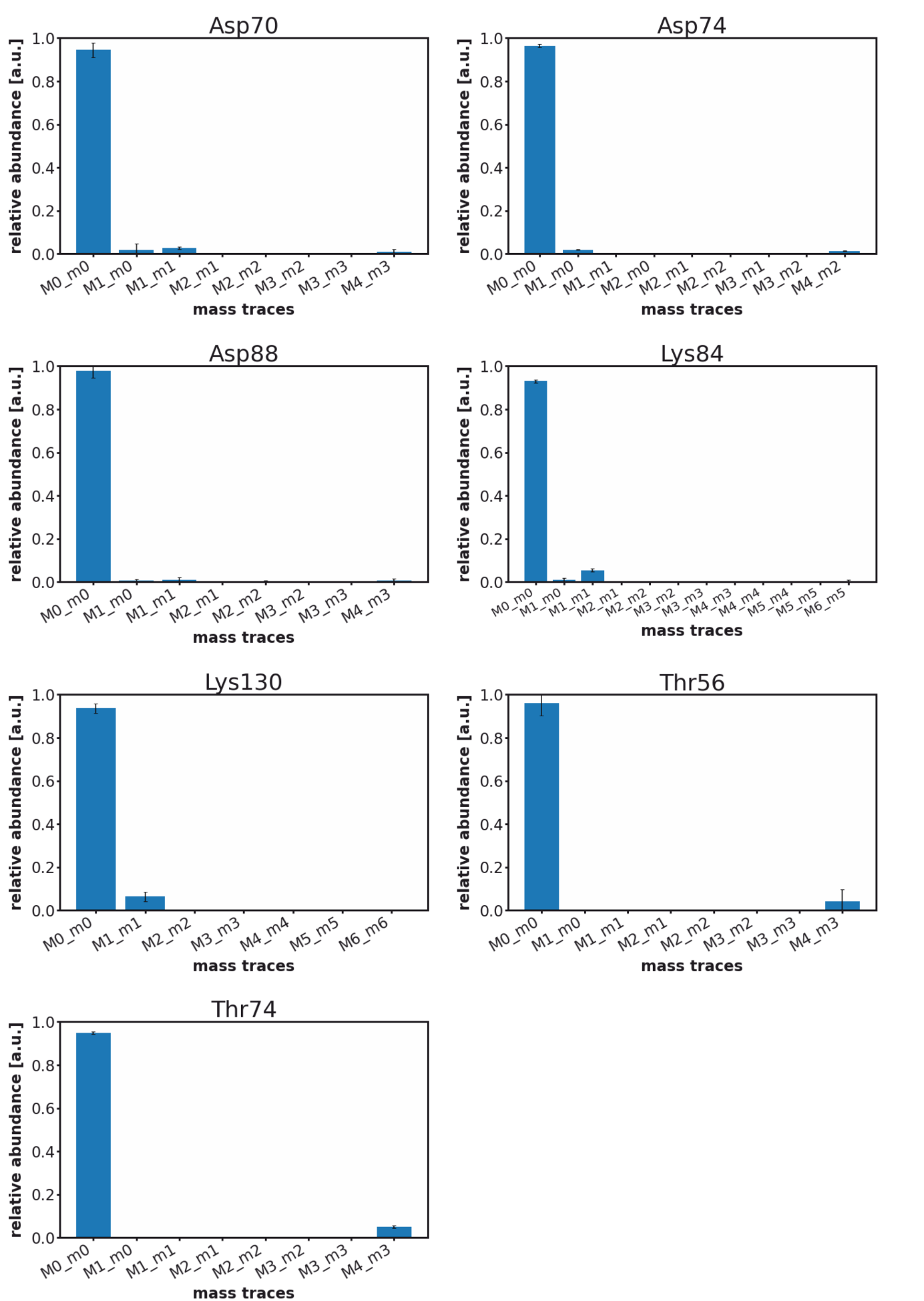


**Figure S11.** Average TMIDs across twelve biological replicates resulting from the spiking experiment in open wells. Here, the measured amino acids from the Asp family are portrayed.


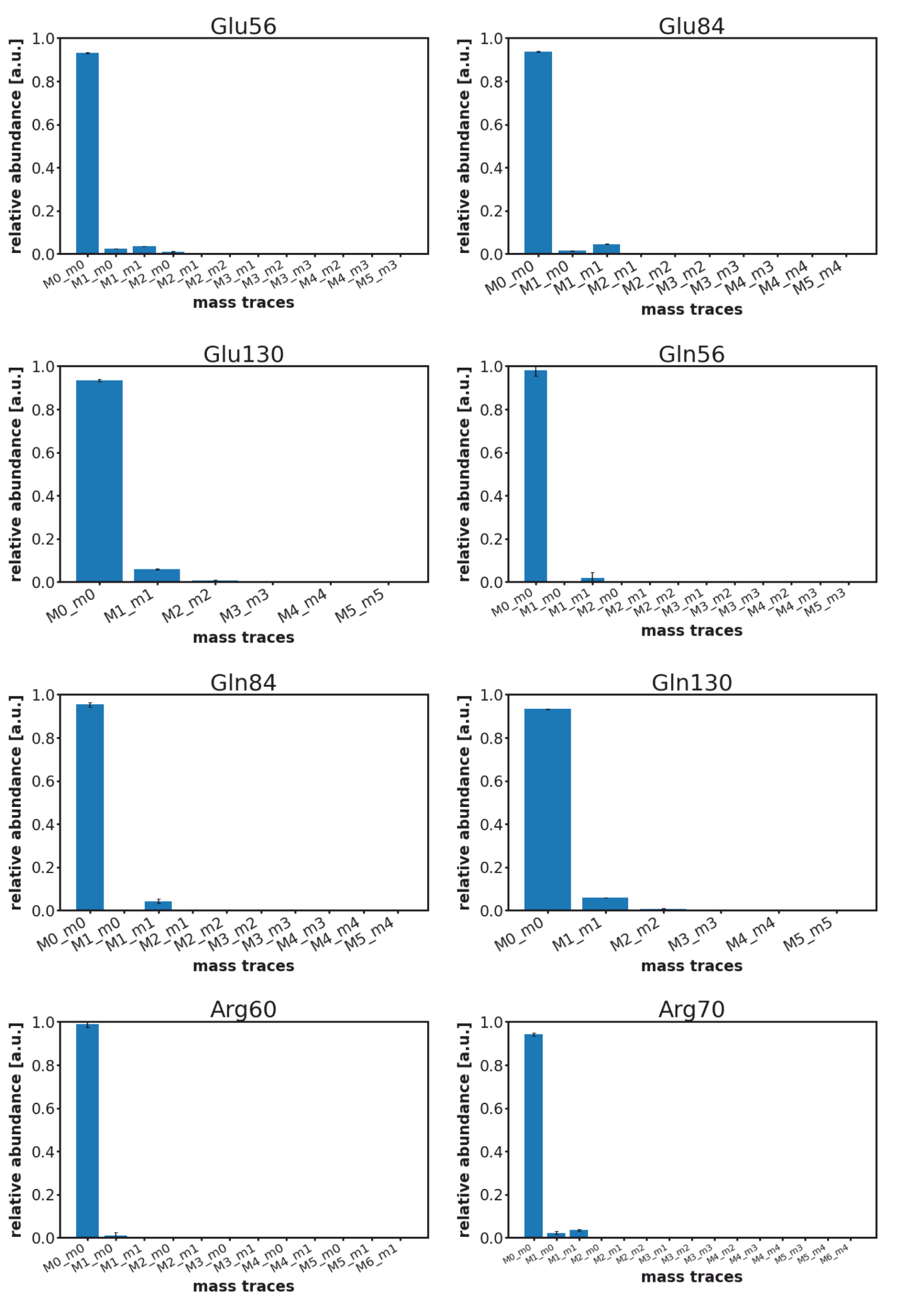


**Figure S12.** Average TMIDs across twelve biological replicates resulting from the spiking experiment in open wells. Here, Glu, Gln, and Arg from the Glu family of amino acids are portrayed.


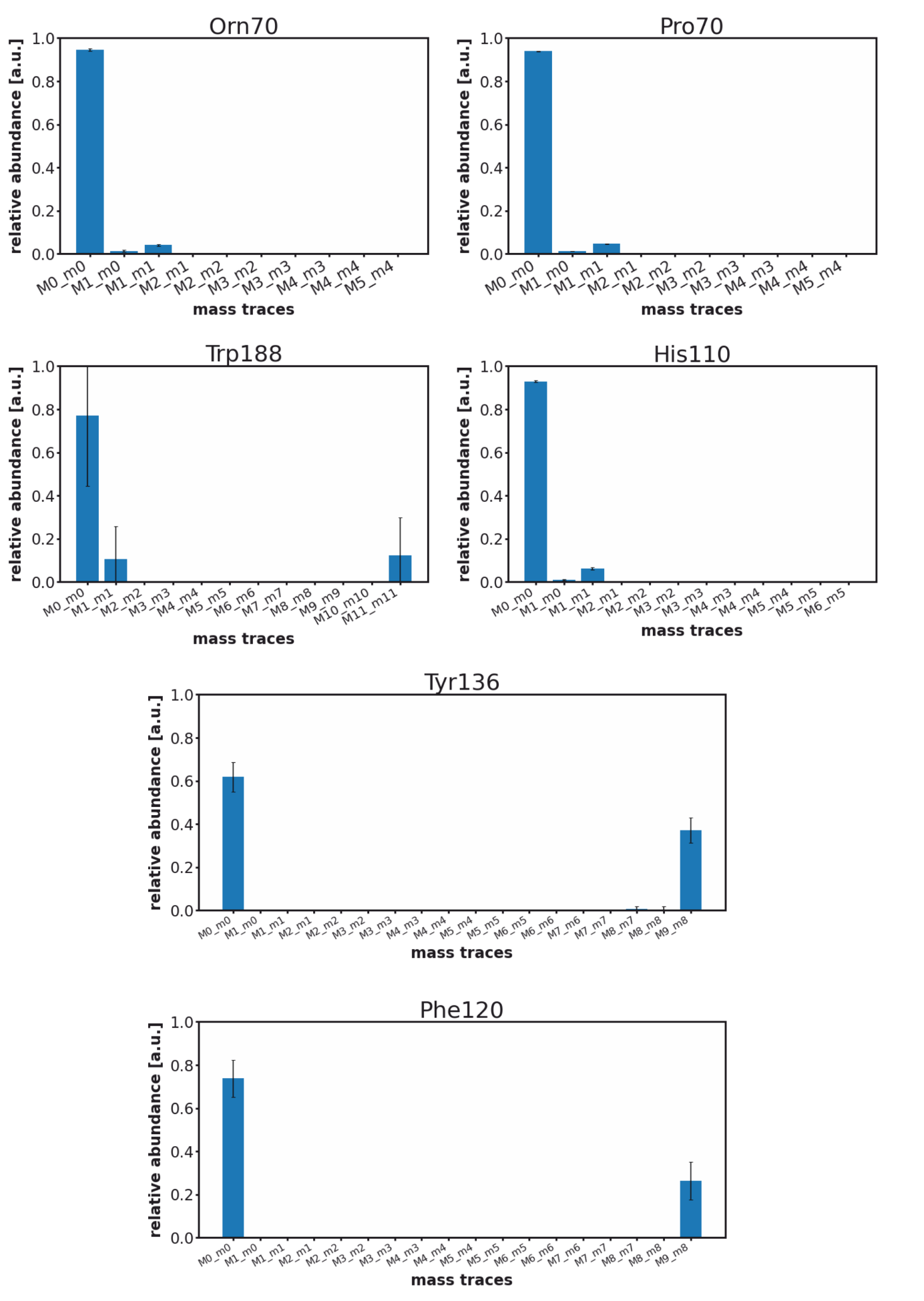


**Figure S13.** Average TMIDs across twelve biological replicates resulting from the spiking experiment in open wells. Here, Orn and Pro from the Glu family, His, and all measured amino acids from the aromatic family are portrayed.

## **
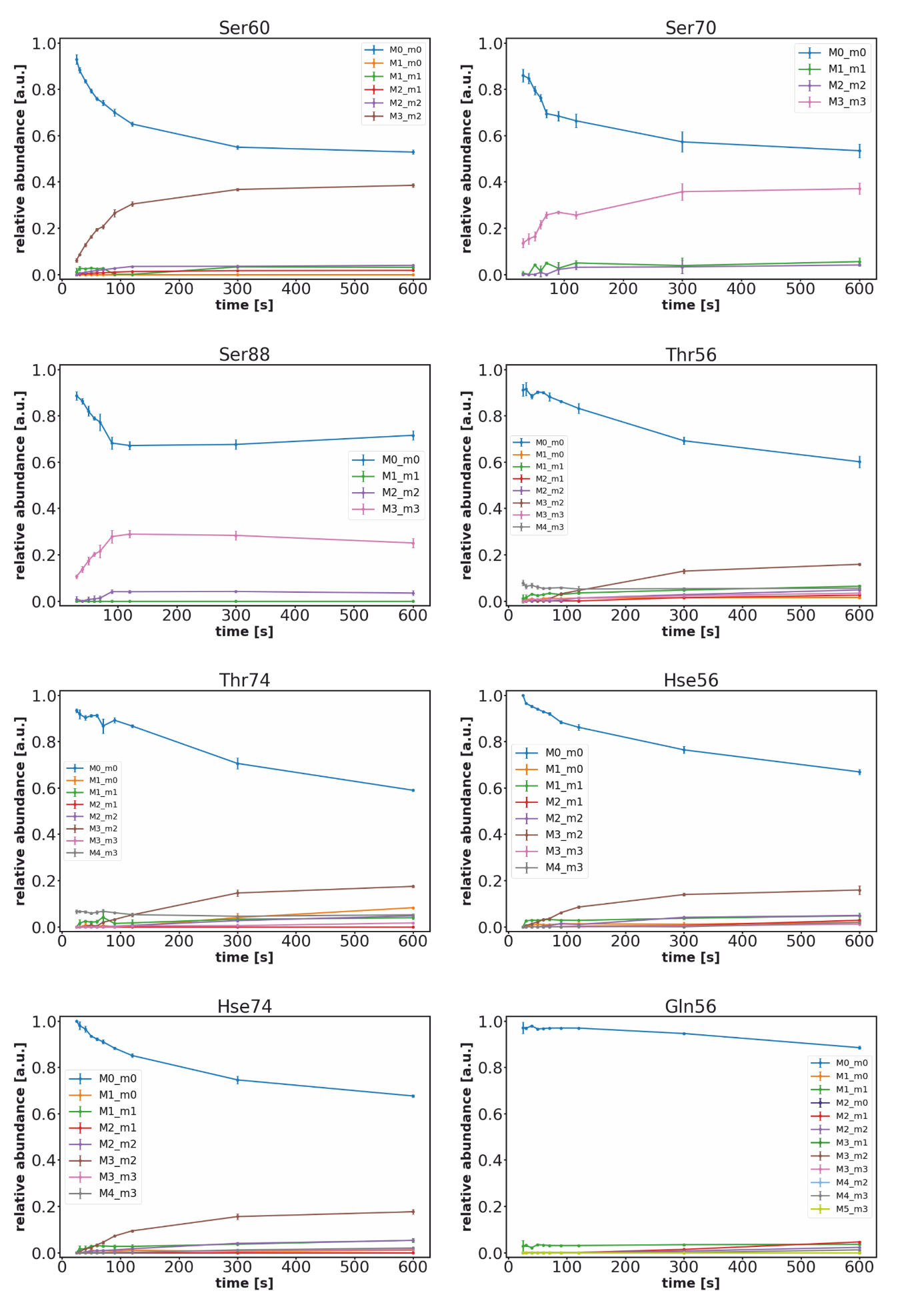
Automated isotopically instationary ILE**

**Figure S14.** Isotopically instationary labeling data of fragments from amino acids with polar side chains Ser, Thr, Hse, and Gln across three biological replicates each.


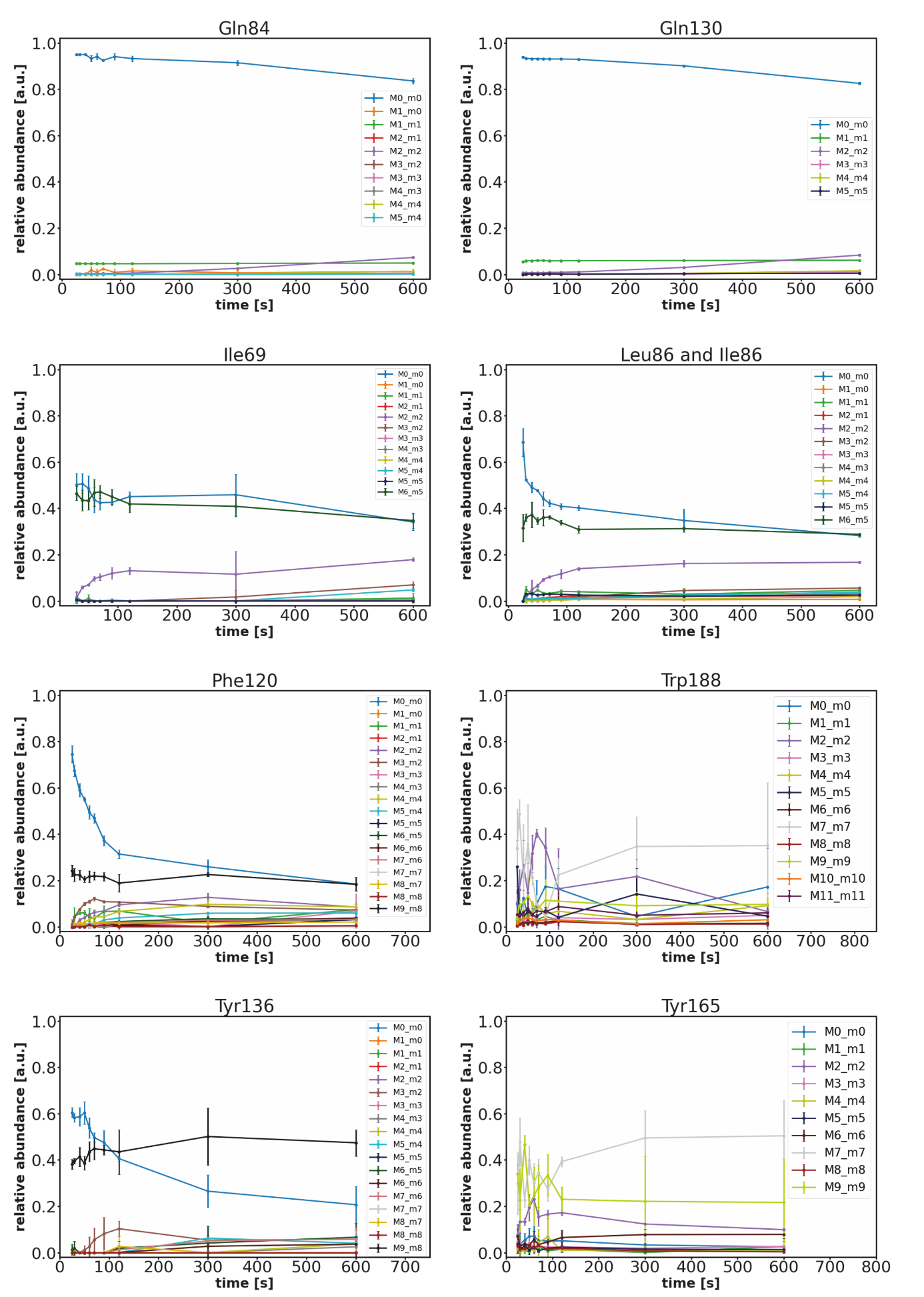
**Figure S15.** Isotopically transient labeling data of fragments from Gln and aromatic amino acids Phe, Trp, and Tyr across three biological replicates each. The leucine and isoleucine peaks were not separated in this LC‑MS/MS run so they were treated as a combined pool.


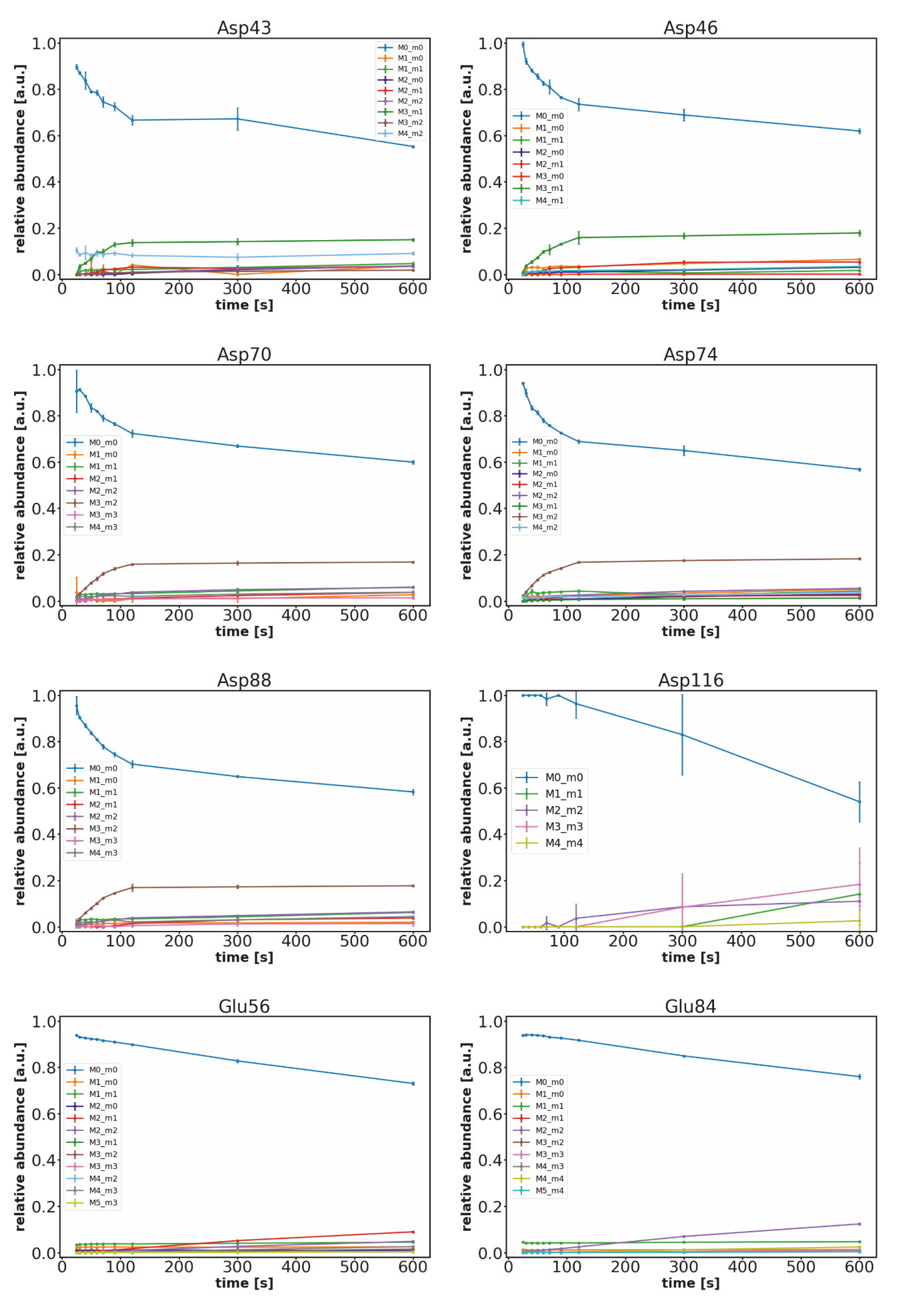
**Figure S16.** Isotopically transient labeling data of fragments from the acidic amino acids Asp and Glu across three biological replicates each.


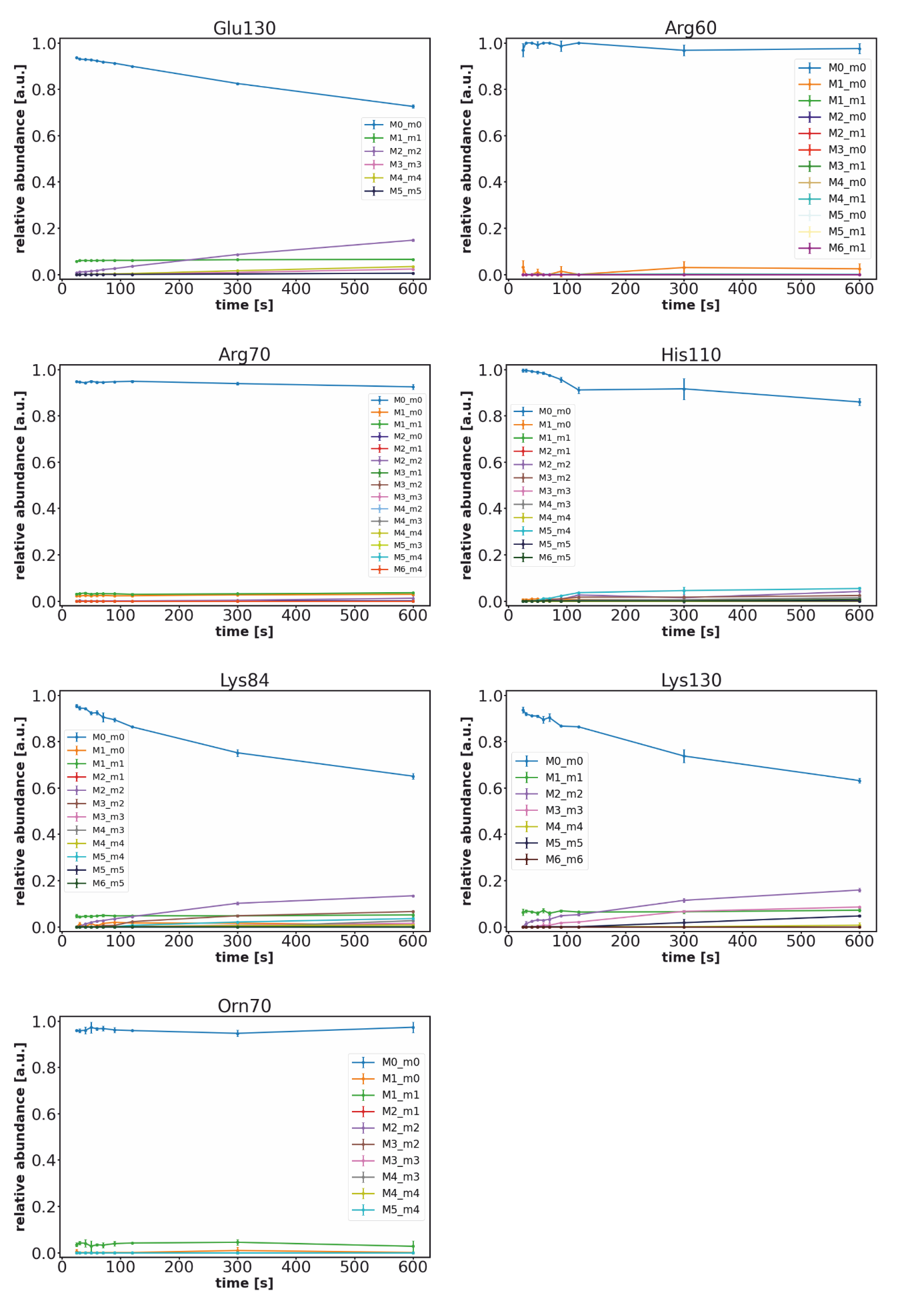
**Figure S17.** Isotopically transient labeling data of fragments from Glu and the basic amino acids Arg, His, Lys, and Orn across three biological replicates each.

**
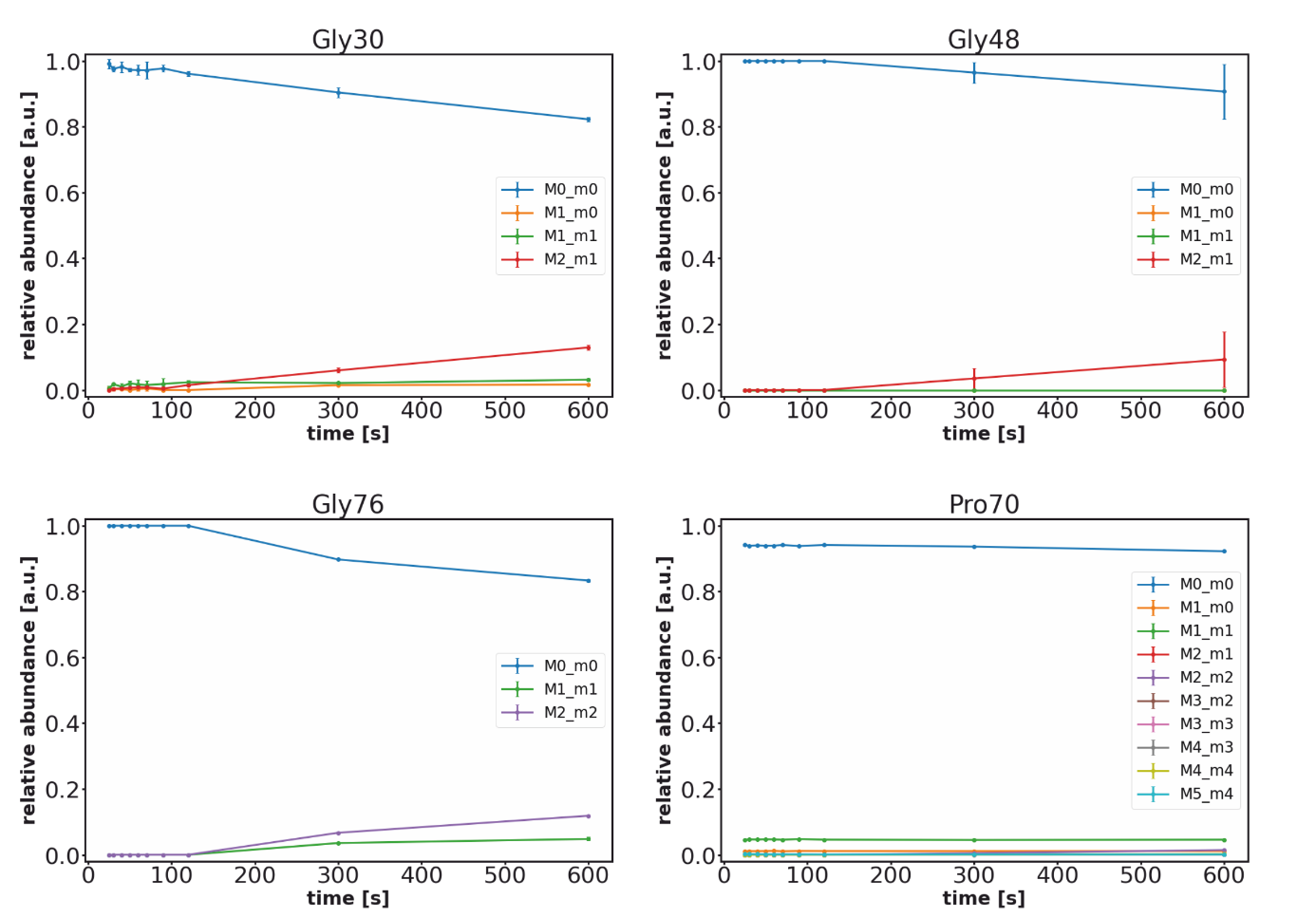
Figure S18.** Isotopically transient labeling data of fragments from the small amino acids Gly and Pro.
